# Serum Proteome Changes in Healthy Subjects with Different Genotypes of *NOS1AP* in the Chinese Population

**DOI:** 10.1155/2013/357630

**Published:** 2013-04-07

**Authors:** Feng Jiang, Congrong Wang, Rongxia Li, Quanhu Sheng, Cheng Hu, Rong Zhang, Qichen Fang, Yuqian Bao, Kunsan Xiang, Rong Zeng, Weiping Jia

**Affiliations:** ^1^Department of Endocrinology and Metabolism, Shanghai Jiao Tong University Affiliated Sixth People's Hospital, Shanghai Diabetes Institute, Shanghai Key Laboratory of Diabetes Mellitus, Shanghai Clinical Center for Diabetes, 600 Yishan Road, Shanghai 200233, China; ^2^Key Laboratory of Systems Biology, Institute of Biochemistry and Cell Biology, Shanghai Institutes for Biological Sciences, Chinese Academy of Sciences, Shanghai 200233, China

## Abstract

Type 2 diabetes and its chronic complications have become a worldwide epidemic nowadays. However, its molecular mechanism is still unknown. We have previously identified a novel variant rs12742393 of *NOS1AP* for type 2 diabetes susceptibility in the Chinese population. In this study, we analyzed the total serum profiling among three genotypes of rs12742393 to discover potential crosstalk under the variant and the disease through proteomic analyses for the first time. We used OFFGEL peptide fractionation, LC-MS/MS analysis, and label-free quantification to profile the fasting human serum samples of the genotypes in rs12742393 (*n* = 4, for CC, AC, and AA, resp.). Four proteins were identified, including apoA4, alpha1-ACT, HABP2, and keratin 10, with blood levels changed significantly between CC and AA homozygotes of rs12742393. Compared with AA group, the levels of apoA4 increased (*P* = 0.000265), whereas the concentration of alpha1-ACT, HABP2, and keratin 10 decreased in CC group (*P* = 0.011116, 0.021175, and 0.015661, resp.). Then we selected additional fasting serum samples for ELISA and western blot validation. However, no significant differences were identified by neither ELISA nor western blot (*P* > 0.05). The protein profiling changes between the genotypes of rs12742393 indicated that this SNP might play a role in the development of type 2 diabetes.

## 1. Introduction

Nitric oxide synthase 1 adaptor protein (NOS1AP), also named as CAPON, regulates the neuronal nitric oxide synthase (nNOS) activity and has an effect on nitric oxide (NO) release by binding N-methyl-d-aspartate receptors (NMDARs) [1]. Recent studies have shown that nNOS is also localized on insulin secreted granules in addition to neuronal tissues and can be activated by increasing intracellular calcium which is a known response to glucose stimulation on *β* cells [2, 3]. Several studies have suggested that both nNOS and NO are directly involved in insulin secretion as well as insulin resistance [4–7]. It was indicated that the interaction between nNOS and glucokinase (GCK) can affect GCK localization and activity and thus influenced glucose-stimulated insulin secretion (GSIS) in cultured *β* cells [4]. Furthermore, a novel mechanism for *β*-cell dysfunction has also been described in which nNOS, as a key protein linking cholesterol and glucose metabolism, can be dimerized to impair GCK activity and reduce GSIS on the insulin granules [8]. In addition, genetic studies have implied that the variations in *NOS1AP* are associated with reduced glucose lowering effect in sulfonylurea users as well as increased incidence of type 2 diabetes in patients taking calcium channel blockers [9, 10]. Though the studies on how the variants influenced the diseases were limited, one functional study showed that rs12742393 could affect *NOS1AP* gene expression through influencing transcription factor binding [11]. Our previous study showed evidence that rs12742393 in *NOS1AP* was involved in type 2 diabetes susceptibility in the Chinese population, with C allele as the risk allele (OR 1.17, 95% CI 1.07–1.26; *P* = 0.0005) [12]. However, the association was not replicated in the European descent [13].

Recently, with the development of genomics and bioinformatics, proteome is widely used to describe all the proteins as well as their various modifications regarding the impact of environment and other stimuli within the whole body. Proteomics allows global screening of complex samples and provides qualitative and quantitative evidence for altered protein expression. Based on the information and initial data, we hypothesized that rs12742393 of *NOS1AP *might have an effect on the development of diabetes in the Chinese population. To test this hypothesis, we investigate the different protein profiling according to the different genotypes (AA, AC, and CC) of rs12742393 by proteomics technology.

## 2. Materials and Methods

### 2.1. Clinical Sample Collection and Preparation

Twelve healthy participants with normal glucose regulation were selected for proteomic investigation, including four CC homozygote, four AC heterozygote, and four AA homozygote individuals. However, we just selected CC and AA carriers for the final statistical analysis and validation, since they can be divided into two distinct groups based on the HCA and PCA. All the individuals for the proteomic analysis were matched strictly with age, sex, BMI, glucose, and lipid related parameters (HbA1c, fasting plasma glucose, OGTT-2 h glucose, triglyceride, total cholesterol, low density lipoprotein, and high density lipoprotein). Then forty-eight healthy participants with twenty-four CC homozygote and twenty-four AA homozygote carriers were included for western blot as well as ELISA validation. All these validation samples were matched with age, BMI, blood glucose, and lipid levels. Serum samples were collected in fasting state and then stored at −20°C before the analysis. The study was approved by the institutional review board of Shanghai Jiao Tong University Affiliated Sixth People's Hospital. Written informed consent was obtained from each participant.

### 2.2. In-Solution Digestion and Peptide OFFGEL Fractionation

The crude plasma was diluted and treated by the albumin-removal manipulation with some modifications to collect plasma proteins [14, 15]. Then the protein mixtures were handled as described to obtain the total peptide mixtures for identification [16]. For p*I*-based peptide separation, we used the 3100 OFFGEL Fractionator (Agilent Technologies, Böblingen, Germany) with a 12-well setup. Electrofocusing of the peptides is performed at 20°C and 50 *μ*A until the 100 kVh level was reached. All fractions were evaporated by centrifugation under vacuum and maintained at −20°C. Prior to MS analysis, samples were desalted by Empore C18 47 mm Disk (3 M).

### 2.3. Label-Free Shotgun Proteomic Identification

Each fractionated peptide was dissolved in 20 *μ*L 0.1% formic acid and loaded into the RP column. RP-HPLC was performed using an Agilent 1100 Capillary system (Agilent Technologies) with C18 column (150 *μ*m i.d., 100 mm length, Column Technology Inc., Fremont, CA). The mass spectral data were acquired on a LTQ linear ion trap mass spectrometer (Thermo, San Jose, CA) equipped with an electrospray interface operated in positive ion mode. The mass spectrometer was set as one full MS scan was followed by ten MS/MS scans on the ten most intense ions from the MS spectrum with the following dynamic exclusion settings: repeat count, 2, repeat duration, 0.5 min, and exclusion duration, 1.5 min.

### 2.4. Database Searching and Protein Identification

All MS/MS spectra were searched using MASCOT algorithm against the human International Protein Index (IPI) database (version 3.73), in which each genuine protein sequence was followed by a reversed amino acid sequence. Carbamidomethylation (57.0214 Da) was searched as a fixed modification on cysteine, and oxidation (15.9949 Da) was set as a variable modification on methionine. Only one missing cleavage site was allowed. All output results were combined together using the in-house software named BuildSummary to delete the redundant data. Searches were conducted against the Human International Protein Index protein sequence database to control the false discovery rate at 1% and all spectral peptide count had a ΔCn score of at least 0.1. Finally, the NSFC (normalized spectral abundance factors) score was calculated for representing the abundance of each protein in the serum [17].

### 2.5. Western Blot Analysis of Four Proteins

We additionally collected forty-eight serum samples with twenty-four CC homozygote and twenty-four AA homozygote carriers for western blot validation. 2 uL of each individual serum sample diluted to 1/40 with 2*SDS was subjected to PAGE-gel electrophoresis, and then proteins in the gel were transferred to a nitrocellulose membrane (Whatman International Ltd., England). The membranes were incubated first with the appropriate primary antibodies overnight at 4°C (apolipoprotein A4 mouse mAb, #5700 from cell signaling technology; keratin 10 rabbit polyclonal Ab, ab97764 from Abcam Ltd., Cambridge, MA; antialpha1-antichymotrypsin mouse mAb, LF-MA0166, AbFrontier; anti-HABP2 rabbit polyclonal Ab, ab81490 from Abcam Ltd., Cambridge, MA, resp.) and then incubated with HRP-conjugated secondary antibodies for 1 h. Signals were detected by enhanced chemiluminescence system (ECL-plus, Amersham PharmaciaBiotech). Gray scale of the protein bands was calculated using Image J software. To decrease the system discrepancy, we used GAPDH with 1 : 1000 dilution (GAPDH rabbit mAb, cell signaling 14C10) as the loading control. Relative levels of target protein in the serum were calculated by the proportion of density ratio of sample bands to that of the loading control band.

### 2.6. Enzyme-Linked Immunosorbent Assay (ELISA) Analysis

Human apolipoprotein A4 (apoA4) ELISA kit (Cat. no. KT-50113, Kamiya Biomedical Company) and human alpha1-antichymotrypsin (alpha1-ACT) ELISA kit (Cat. no. KT-498, Kamiya Biomedical Company) were available to quantify the protein levels. Original and 1 : 2500 diluted serum samples were prepared for apoA4 and alpha1-ACT detection, respectively, and then standard procedures were followed by the instructions of each ELISA kit. The absorbance of each sample at 450 nm was recorded using a Bio-Rad microplate reader model.

### 2.7. Statistical Analysis

Data were shown as mean ± standard error (str) for normally distributed values. Differences between groups for normally distributed variables were compared using two-tailed *t*-test. All calculations were performed with SAS (version 8.0; SAS Institute, Cary, NC). A *P* value below 0.05 was considered statistically significant.

## 3. Results

### 3.1. Semiquantitative Proteomic Identification in the Serum

**Figure 1:**
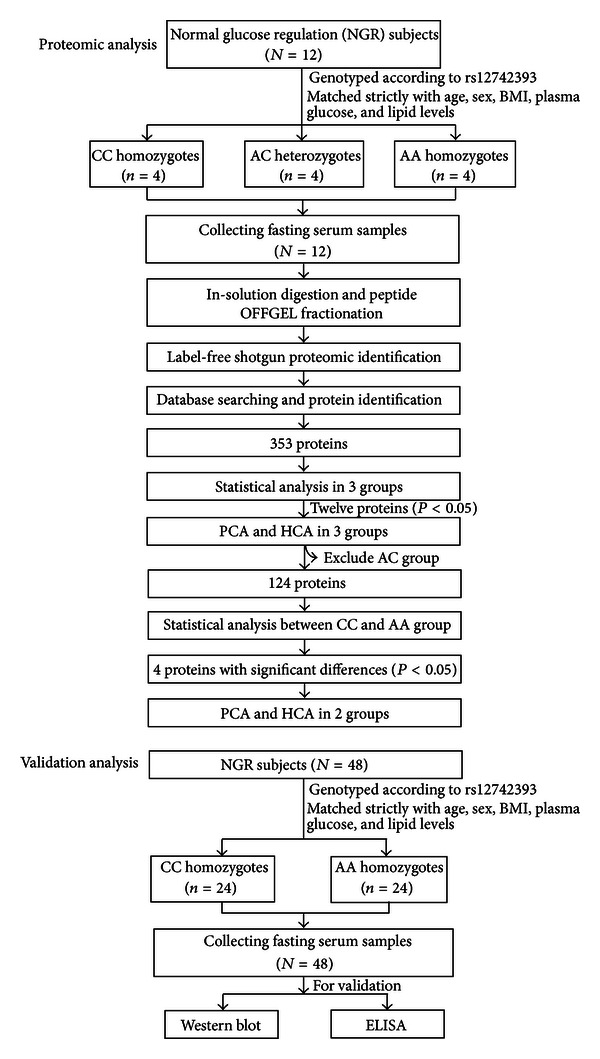
Flow chart of the study.

We analyzed differential protein profile in three groups using shotgun proteomics and label-free quantitative strategy (Figure 1). The proteins were identified with criteria corresponding to an estimated false discovery rate of 1%. After combining the MS/MS data generated from all experiments, 62,523 peptide counts leading to identification of 1,725 unique peptides were assigned to 353 protein groups in twelve serum samples. For semiquantitative analysis, protein identified at least in seven samples was selected in our data. 

### 3.2. HCA and PCA Presentation

**Figure 2:**
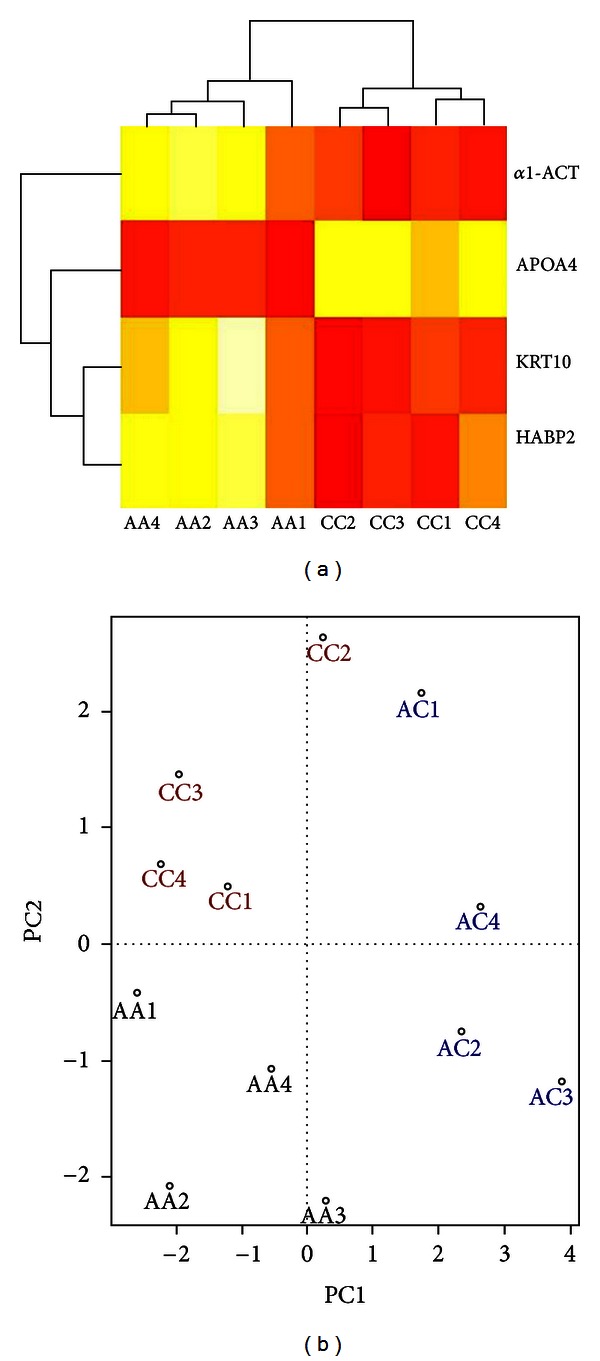
Global relationships were visualized by performing “hierarchical cluster analysis” (HCA (a)) and “principal component analysis” (PCA, (b)) according to the significantly changed proteins. AA1-AA4 and CC1-CC4 represent the eight samples for proteomics analysis.

To visualize the global pattern related to type 2 diabetes, we used HCA and PCA in this study. As shown in Figure 2, HCA and PCA can completely divide the CC and AA carriers into two distinct groups. Therefore, we excluded AC group and only compared the other two groups (CC and AA homozygotes) to investigate the different protein profiling. Finally, 124 proteins were selected for statistical analysis and further validation between CC and AA groups (see Supplementary material (Table 1) available online at http://dx.doi.org/10.1155/2013/357630).

### 3.3. Clinical Data

Twelve subjects were selected for the proteomic analysis, but only eight subjects with four CC carriers and four AA carriers were selected for further validation based on the PCA and HCA results (Table 1). Additional forty-eight samples were selected for western blot and ELISA validation, with twenty-four CC carriers and twenty-four AA carriers (Supplementary Table 2).

### 3.4. Statistical Analysis for Significantly Changed Proteins

To obtain significantly changed proteins related to diabetes, the 124 proteins were ranked based on the quantitative data and showed that four proteins including apolipoprotein A4 (apoA4), alpha1-antichymotrypsin (alpha-1-ACT), keratin 10, and hyaluronan-binding protein 2 (HABP2) had a significant difference (*P* < 0.05) between AA and CC groups. Compared with AA group, the levels of apoA4 increased (*P* = 0.000265), whereas the concentration of alpha1-ACT, keratin 10, and HABP2 decreased in CC group (*P* = 0.011116, 0.015661, and 0.021175, resp.). These four proteins are involved in the lipoprotein metabolism, acute inflammatory response, and epidermis development as well as cell adhesion (Table 2).

### 3.5. Semiquantification Analysis by Western Blot

ApoA4, alpha1-ACT, keratin 10, and HABP2 were validated by western blot in additional serum samples from twenty-four CC homozygotes and twenty-four AA homozygotes. The integrated densities of all the target proteins were normalized by GAPDH. No significant differences were replicated in any of the four proteins by western blot. However, the differences of keratin 10 showed some trend between CC and AA groups (*P* = 0.067).

### 3.6. Quantification Analysis by ELISA

In order to determine the levels of four proteins as identified by LTQ, ELISA was used to analyze human apoA4 and alpha1-ACT levels (ELISA kits available) in expanded serum samples (*n* = 48, the same as used in western blot). All the absorbance was adjusted by the standard blank calibrate sample and the target protein concentrations were calculated according to the formula derived from the standard calibrators. However, no significant differences were observed in either of the two proteins between the two groups.

## 4. Discussion

 In this study, the RP-HPLC-MS/MS coupled with quantitative analysis strategy was applied to investigate the different protein profiling of rs12742393 genotypes in *NOS1AP*. The present study investigated the possible function of *NOS1AP* using proteomic analysis for the first time.

Our data showed an increasing level of the apoA4 in the CC homozygote carriers, while it showed decreasing levels of alpha1-ACT HABP2 as well as keratin 10 compared with AA homozygote carriers. These proteins were involved in lipoprotein metabolism, acute phase of inflammation or infection, and cell proliferation or migration, as well as epidermis development, which might contribute to the development of diabetes.

Apolipoprotein A4 (apoA4), secreted by small intestine in response to fat absorption, contributed a lot to lipid absorption, reverse cholesterol transport [18], anti-inflammatory response, [19] and, particularly, glucose homeostasis. For example, apoA4 had a direct effect on enhancing glucose-stimulated insulin secretion in pancreatic islets. ApoA4 knockout mice also showed impaired glucose tolerance and insulin secretion while exogenous apoA4 administration to apoA4^−/−^ mice could improve glucose tolerance through increasing insulin secretion [20]. Moreover, genetic variants in apoA4 also indicated an association with fasting glucose levels [21]. Therefore, apoA4, the only upregulated protein in the risk allele carriers in our research, might have an association with type 2 diabetes through the interaction with NOS1AP (or nNOS). The mechanism should be elucidated by other functional studies.

Alpha1-antichymotrypsin (alpha1-ACT) is an acute phase protein produced in the liver, which is induced during inflammation response. Elevating levels of alpha1-ACT were observed in the hepatocellular carcinoma patients using 2D-LC MALDI-MS/MS [22]. Moreover, recent studies have confirmed that IL-1*β* could modify IL-6-induced acute phase protein production, such as alpha1-ACT, through a complex intracellular crosstalk between STAT3 and NF-*κ*B-mediated signal transductions [23]. This evidence might provide us some new perspectives to find the internal links between alpha1-ACT and diabetes.

Hyaluronan-binding protein 2 (HABP2) is an HA-binding extracellular serine protease, which is involved in the extrinsic pathway of blood coagulation and fibrinolysis. Although HABP2 is mainly produced in the liver, many studies have demonstrated its expression in pulmonary endothelium cells, which contributed a lot to the lung injury [24–27]. Up till now, no direct evidence has linked HABP2 with diabetes; however, its ligand, hyaluronan, has been suggested to interact with CD44 and PKC and reduce inflammation in diabetic nephropathy mice [28]. Our data showed a decreased HABP2 level in CC carriers, which might give us some hints to find the crosstalk between this protein and diabetes.

Keratin 10 is a member of the type I (acidic) cytokeratin family, which belongs to the superfamily of intermediate filament (IF) proteins. Due to its classic role in the epidermis development, mutations in *KRT10* were associated with epidermolytic hyperkeratosis [29, 30]. However, keratin 10 also had an interaction with both protein kinase B (PKB) and atypical PKC*δ*, which could impair the activation of the kinase and inhibit their intracellular translocation [31]. As we know, PKB and PKC pathways could regulate glucose homeostasis; thus, the impairment of PKB and PKC*δ* by keratin 10 might induce potential metabolic disorders [32, 33]. Further research should be performed to elucidate the mechanism.

Overall, it was the first time to investigate the potential function of susceptible variant to type 2 diabetes using proteomic technology. We found four proteins that might associate with the mechanism of diabetes. However, the intrinsic interaction between these proteins and NOS1AP and the mechanism by which rs12742393 in *NOS1AP* mediated still need further research to elucidate. In addition, the results of proteomic analysis were not replicated in western blot or ELISA in the expanded samples; thus, we could not totally exclude the “false positive” of proteomic analysis. The reasons that we did not validate the results in neither western blot nor ELISA were probably due to the minor differences in clinical characteristics between the two sets of subjects (subjects for proteomic analysis and for validation analysis). For example, BMI of the subjects in the proteomic analysis (mean BMI > 26 kg/m^2^) is higher than that in the validation analysis (mean BMI < 24 kg/m^2^); thus, it might have an effect on the glucose and lipid metabolism potentially. In addition, sex should be also taken into consideration in the analysis. Therefore, we should match the glucose and lipid related traits more strictly to exclude the interpretation besides increasing the sample size. However, as what we described in the method, the quality controls of the proteomic analysis were strict; thus, we believed that the proteins that showed significant differences between the two groups were not just an incidental result. Therefore, it might be due to the different characteristics between the two sets of subjects and limited samples in the validation analysis. More samples should be involved in the study to perform the validation test.

We detected four proteins showing significant differences between CC and AA carriers of rs12742393 in *NOS1AP*. Although we failed to validate these differences in the large sample cohort, we suggested that these four proteins might associate with the development of type 2 diabetes in subgroup of patients through the crosstalk with NOS1AP protein, which might provide us a new perspective to the mechanism of type 2 diabetes.

## Supplementary Material

Supplementary table 1 showed the detailed information of the 124 proteins after proteomic analysis. All MS/MS spectra were searched according to the human International Protein Index (IPI) database (version 3.73). In addition, the abundance of each protein in the serum was represented with NSFC (normalized spectral abundance factors) score. Supplementary table 2 showed clinical characteristics of the CC and AA carriers for western blot as well as ELISA validation. Subjects in these two groups were matched with age, BMI, glucose and lipid related parameters.

## Figures and Tables

**Table 1:** Clinical characteristics of the four CC carriers and four AA carriers.

	CC genotype (risk allele)	AA genotype (nonrisk allele)	*P* value
Male/female	4/0	4/0	>0.05
Age	54.0 ± 4.06	54.25 ± 0.48	0.9550
BMI (kg/m^2^)	26.28 ± 0.83	26.38 ± 0.56	0.9244
FPG (mmol/L)	5.01 ± 0.15	5.01 ± 0.17	0.9831
2 h glucose (mmol/L)	5.32 ± 0.33	5.68 ± 0.44	0.5413
HbA1c (%)	5.7 ± 0.12	5.8 ± 0.29	0.7620
TC (mmol/L)	4.98 ± 0.40	5.0 ± 0.38	0.9652
TG (mmol/L)	1.74 ± 0.39	2.63 ± 0.28	0.1136
HDL (mmol/L)	1.01 ± 0.11	1.10 ± 0.07	0.5329
LDL (mmol/L)	3.23 ± 0.31	3.24 ± 0.35	0.9919

Data were shown as mean ± str.

FPG: fasting plasma glucose; HbA1c: glycated hemoglobin A1c; TC: total cholesterol; TG: triglyceride; HDL: high density lipoprotein; LDL: low density lipoprotein.

**Table 2:** Identification of proteins with significant differences between CC carriers and AA carriers based on NSAF value.

IPI ID	Protein name	CC carriers mean NSAF	AA carriers mean NSAF	*P* value
00009865.4	Keratin10	−8.038	−6.938	0.015661
00847635.1	Alpha-1-antichymotrypsin	−6.037	−5.501	0.011116
00304273.2	Apolipoprotein A4	−8.339	−11.107	0.000265
00746623.2	Hyaluronan-binding protein 2	−9.435	−8.623	0.021175

NSAF: normalized spectral abundance factors.

## References

[1] Jaffrey SR, Snowman AM, Eliasson MJL, Cohen NA, Snyder SH (1998). CAPON: a protein associated with neuronal nitric oxide synthase that regulates its interactions with PSD95. *Neuron*.

[2] Aspinwall CA, Qian WJ, Roper MG, Kulkarni RN, Kahn CR, Kennedy RT (2000). Roles of insulin receptor substrate-1, phosphatidylinositol 3-kinase, and release of intracellular Ca^2+^ stores in insulin-stimulated insulin secretion in *β*-cells. *Journal of Biological Chemistry*.

[3] Lajoix AD, Reggio H, Chardès T (2001). A neuronal isoform of nitric oxide synthase expressed in pancreatic *β*-cells controls insulin secretion. *Diabetes*.

[4] Rizzo MA, Piston DW (2003). Regulation of *β* cell glucokinase by S-nitrosylation and association with nitric oxide synthase. *Journal of Cell Biology*.

[5] Shankar R, Zhu JS, Ladd B, Henry D, Shen HQ, Baron AD (1998). Central nervous system nitric oxide synthase activity regulates insulin secretion and insulin action. *Journal of Clinical Investigation*.

[6] Shankar RR, Wu Y, Shen HQ, Zhu JS, Baron AD (2000). Mice with gene disruption of both endothelial and neuronal nitric oxide synthase exhibit insulin resistance. *Diabetes*.

[7] Smukler SR, Tang L, Wheeler MB, Salapatek AMF (2002). Exogenous nitric oxide and endogenous glucose-stimulated *β*-cell nitric oxide augment insulin release. *Diabetes*.

[8] Hao M, Head WS, Gunawardana SC, Hasty AH, Piston DW (2007). Direct effect of cholesterol on insulin secretion: a novel mechanism for pancreatic *β*-cell dysfunction. *Diabetes*.

[9] Becker ML, Aarnoudse AJLHJ, Newton-Cheh C (2008). Common variation in the NOS1AP gene is associated with reduced glucose-lowering effect and with increased mortality in users of sulfonylurea. *Pharmacogenetics and Genomics*.

[10] Becker ML, Visser LE, Newton-Cheh C (2008). Genetic variation in the NOS1AP gene is associated with the incidence of diabetes mellitus in users of calcium channel blockers. *Diabetologia*.

[11] Wratten NS, Memoli H, Huang Y (2009). Identification of a schizophrenia-associated functional noncoding variant in NOS1AP. *American Journal of Psychiatry*.

[12] Hu C, Wang C, Zhang R (2010). Association of genetic variants of NOS1AP with type 2 diabetes in a Chinese population. *Diabetologia*.

[13] Prokopenko I, Zeggini E, Hanson RL (2009). Linkage disequilibrium mapping of the replicated type 2 diabetes linkage signal on chromosome 1q. *Diabetes*.

[14] Fu Q, Garnham CP, Elliott ST, Bovenkamp DE, van Eyk JE (2005). A robust, streamlined, and reproducible method for proteomic analysis of serum by delipidation, albumin and IgG depletion, and two-dimensional gel electrophoresis. *Proteomics*.

[15] Gundry RL, Fu Q, Jelinek CA, van Eyk JE, Cotter RJ (2007). Investigation of an albumin-enriched fraction of human serum and its albuminome. *Proteomics*.

[16] Li RX, Zhou H, Li SJ, Sheng QH, Xia QC, Zeng R (2005). Prefractionation of proteome by liquid isoelectric focusing prior to two-dimensional liquid chromatography mass spectrometric identification. *Journal of Proteome Research*.

[17] Zybailov B, Mosley AL, Sardiu ME, Coleman MK, Florens L, Washburn MP (2006). Statistical analysis of membrane proteome expression changes in Saccharomyces cerevisiae. *Journal of Proteome Research*.

[18] Stan S, Delvin E, Lambert M, Seidman E, Levy E (2003). Apo A-IV: an update on regulation and physiologic functions. *Biochimica et Biophysica Acta*.

[19] Vowinkel T, Mori M, Krieglstein CF (2004). Apolipoprotein A-IV inhibits experimental colitis. *Journal of Clinical Investigation*.

[20] Wang F, Kohan AB, Kindel TL (2012). Apolipoprotein A-IV improves glucose homeostasis by enhancing insulin secretion. *Proceedings of the National Academy of Sciences of the United States of America*.

[21] Larson IA, Ordovas JM, Sun Z (2002). Effects of apolipoprotein A-IV genotype on glucose and plasma lipoprotein levels. *Clinical Genetics*.

[22] Ishihara T, Fukuda I, Morita A (2011). Development of quantitative plasma N-glycoproteomics using label-free 2-D LC-MALDI MS and its applicability for biomarker discovery in hepatocellular carcinoma. *Journal of Proteomics*.

[23] Bode JG, Albrecht U, Haussinger D, Heinrich PC, Schaper F (2012). Hepatic acute phase proteins—regulation by IL-6- and IL-1-type cytokines involving STAT3 and its crosstalk with NF-kappaB-dependent signaling. *European Journal of Cell Biology*.

[24] Choi-Miura NH, Tobe T, Sumiya JI (1996). Purification and characterization of a novel hyaluronan-binding protein (PHBP) from human plasma: it has three EGF, a kringle and a serine protease domain, similar to hepatocyte growth factor activator. *Journal of Biochemistry*.

[25] Kannemeier C, Feussner A, Stöhr HA, Weisse J, Preissner KT, Römisch J (2001). Factor VII and single-chain plasminogen activator-activating protease: activation and autoactivation of the proenzyme. *European Journal of Biochemistry*.

[26] Kanse SM, Parahuleva M, Muhl L, Kemkes-Matthes B, Sedding D, Preissner KT (2008). Factor VII-activating protease (FSAP): vascular functions and role in atherosclerosis. *Thrombosis and Haemostasis*.

[27] Mambetsariev N, Mirzapoiazova T, Mambetsariev B (2010). Hyaluronic acid binding protein 2 is a novel regulator of vascular integrity. *Arteriosclerosis, Thrombosis, and Vascular Biology*.

[28] Campo GM, Avenoso A, Micali A (2010). High-molecular weight hyaluronan reduced renal PKC activation in genetically diabetic mice. *Biochimica et Biophysica Acta*.

[29] Arin MJ, Longley MA, Anton-Lamprecht I (1999). A novel substitution in keratin 10 in epidermolytic hyperkeratosis. *Journal of Investigative Dermatology*.

[30] Sun XK, Ma LL, Xie YQ, Zhu XJ (2002). Keratin 1 and keratin 10 mutations causing epidermolytic hyperkeratosis in Chinese patients. *Journal of Dermatological Science*.

[31] Paramio JM, Segrelles C, Ruiz S, Jorcano JL (2001). Inhibition of protein kinase B (PKB) and PKC*ζ* mediates keratin K10-induced cell cycle arrest. *Molecular and Cellular Biology*.

[32] de Marinis YZ, Zhang E, Amisten S (2010). Enhancement of glucagon secretion in mouse and human pancreatic alpha cells by protein kinase C (PKC) involves intracellular trafficking of PKC*α* and PKC*δ*. *Diabetologia*.

[33] Lima CR, Vasconcelos CF, Costa-Silva JH (2012). Anti-diabetic activity of extract from Persea americana Mill. leaf via the activation of protein kinase B, (PKB/Akt) in streptozotocin-induced diabetic rats. *Journal of Ethnopharmacology*.

