# Supplementary material for: Serum Proteome Changes in Healthy Subjects with Different Genotypes of NOS1AP in the Chinese Population
Source: J Diabetes Res. 2013 Apr 7;2013:357630. doi: 10.1155/2013/357630 (PMC3647583; doi:10.1155/2013/357630)
Supplement: Supplementary file 1 — Supplementary table 1 showed the detailed information of the 124 proteins after proteomic analysis. All MS/MS spectra were searched according to the human International Protein Index (IPI) database (version 3.73). In addition, the abundance of each protein in the serum was represented with NSFC (normalized spectral abundance factors) score. Supplementary table 2 showed clinical characteristics of the CC and AA carriers for western blot as well as ELISA validation. Subjects in these two groups were matched with age, BMI, glucose and lipid related parameters. [file 357630.f1.pdf]

Protein	Description	1	2	3	4	5	6	8	9	12	13	14	15	1_count	2_count	3_count	4_count	5_count	6_count	8_count	9_count	12_count	13_count	14_count	15_count
IPI00022229.1	Tax_Id=9606 Gene_Symbol=APOB Apolipoprotein B-100	-6.870116	-6.615649	-6.762898	-6.715934	-6.903232	-6.656584	-6.266894	-6.561369	-6.878192	-6.53119	-6.301183	-6.70611	217	224	229	227	257	154	253	212	221	126	186	148
IPI00783987.2	Tax_Id=9606 Gene_Symbol=C3 Complement C3 (Fragment)	-5.776825	-5.354977	-5.736225	-5.671941	-5.492309	-5.318203	-4.918808	-5.367334	-5.807412	-5.554094	-5.554728	-5.663527	236	288	233	235	384	214	355	255	235	122	143	153
IPI00643525.1	Tax_Id=9606 Gene_Symbol=C4A Putative uncharacterized protein C4A	-6.467933	-6.522201	-6.422637	-6.383294	-6.378522	-6.031257	-5.602042	-6.283327	-6.750207	-6.454429	-6.170681	-6.384815	124	94	123	121	166	110	188	107	96	52	81	78
IPI00892604.1	Tax_Id=9606 Gene_Symbol=C4A;LOC100292046;C4B Complement component C4B (Childo blood group) 2	-6.459901	-6.490785	-6.422637	-6.391593	-6.354711	-6.013238	-5.596737	-6.283327	-6.77126	-6.454429	-6.170681	-6.397718	125	97	123	120	170	112	189	107	94	52	81	77
IPI00654875.1	Tax_Id=9606 Gene_Symbol=C4B Complement C4-B	-6.47603	-6.480529	-6.455698	-6.4084	-6.372516	-6.058908	-5.639987	-6.350986	-6.77126	-6.454429	-6.208422	-6.437458	123	98	119	118	167	107	181	100	94	52	78	74
IPI00032258.4	Tax_Id=9606 Gene_Symbol=C4A Complement C4-A	-6.492424	-6.511619	-6.455698	-6.399961	-6.396759	-6.077777	-5.645527	-6.350986	-6.750207	-6.454429	-6.208422	-6.424035	121	95	119	119	163	105	180	100	96	52	78	75
IPI00478003.2	Tax_Id=9606 Gene_Symbol=A2M Alpha-2-macroglobulin	-5.782475	-5.104281	-5.924956	-5.599237	-5.258523	-5.421872	-5.366978	-5.525264	-6.010555	-5.806655	-5.669541	-5.78583	208	328	171	224	430	171	201	193	170	84	113	120
IPI00845263.1	Tax_Id=9606 Gene_Symbol=FN1 fibronectin isoform 2 preproprotein	-7.457331	-7.404511	-7.388433	-7.464033	-7.487775	-7.370857	-6.822678	-7.313863	-7.408448	-7.150154	-6.673622	-7.026472	64	54	65	57	76	40	77	53	69	36	68	57
IPI00414283.6	Tax_Id=9606 Gene_Symbol=FN1 Isoform 9 of Fibronectin	-7.411375	-7.345499	-7.310729	-7.368936	-7.423316	-7.293152	-6.744973	-7.236159	-7.330743	-7.072449	-6.595917	-6.948767	62	53	65	58	75	40	77	53	69	36	68	57
IPI00479723.4	Tax_Id=9606 Gene_Symbol=FN1 FN1 protein	-7.379626	-7.345499	-7.341981	-7.368936	-7.410071	-7.31847	-6.744973	-7.255207	-7.390462	-7.072449	-6.595917	-6.948767	64	53	63	58	76	39	77	52	65	36	68	57
IPI00745872.2	Tax_Id=9606 Gene_Symbol=ALB Isoform 1 of Serum albumin	-3.069058	-2.814446	-2.557108	-2.640914	-3.318755	-1.861993	-3.056542	-3.11845	-3.181569	-2.841809	-2.97054	-3.370437	1296	1338	2050	1783	1236	2484	837	885	1189	673	694	555
IPI00029739.5	Tax_Id=9606 Gene_Symbol=CFH Isoform 1 of Complement factor H	-6.44005	-6.440475	-6.50444	-6.319871	-6.497764	-6.622183	-5.99032	-6.547359	-7.252629	-6.838443	-6.057893	-												

6.564528	90	72	80	91	104	43	90	58	41	25	64
46
IPI00022463.1	Tax_Id=9606 Gene_Symbol=TF Serotransferrin	-4.777786
-5.145832	-4.789675	-4.86069	-4.419934	-4.192002	-4.346818
-5.010001	-5.305088	-4.753757	-4.555663	-4.49793	269	149
252	222	471	277	264	153	163	114	163	206
IPI00019580.1	Tax_Id=9606 Gene_Symbol=PLG Plasminogen -6.832433	-
7.163099	-6.912571	-6.69861	-7.010035	-6.969103	-6.221434	-
6.181921	-7.081917	-7.441546	-6.579352	-6.755745	40	23	35
41	41	20	47	55	32	9	25	25
IPI00019591.2	"Tax_Id=9606 Gene_Symbol=- cDNA FLJ55673, highly similar
to Complement factor B"	-7.254324	-7.161658	-7.417995	-7.562929
-7.386001	-7.320376	-6.586339	-7.268541	-7.77536	-7.140915
-6.986715	-7.28571	41	36	33	27	44	22	51	29
25	19	26	23
IPI00944960.1	Tax_Id=9606 Gene_Symbol=ITIH4 ITIH4 protein	-6.927155
-6.778544	-6.94787	-6.892133	-7.038478	-6.776143	-6.244802
-6.619194	-7.080247	-6.044613	-6.763687	-6.684146	42	39
39	39	46	28	53	41	37	42	24	31
IPI00032291.2	Tax_Id=9606 Gene_Symbol=C5 Complement C5	-8.304004
-8.192511	-7.899213	-7.738116	-7.985002	-8.746056	-7.663219
-7.450649	-8.279052	-8.28646	-7.58092	-7.523698	19	17
27	30	32	7	23	32	20	8	19	24
IPI00220327.4	"Tax_Id=9606 Gene_Symbol=KRT1 Keratin, type II
cytoskeletal 1"	-6.826241	-7.296669	-6.980487	-6.656486	-6.997764
-5.803674	-6.797724	-6.868876	-7.427946	-5.825916	-5.784703
-6.007616	32	16	26	34	33	51	21	22	18	36
44	42
IPI00025426.3	Tax_Id=9606 Gene_Symbol=PZP Isoform 1 of Pregnancy zone
protein	-8.352837	-8.012335	-8.874808	-7.758199	-7.960425	-
8.266363	-7.785324	-8.395472	-8.060724	-8.85659	-7.629753	-
7.806146	16	18	9	26	29	10	18	11	22	4	16
16
IPI00019568.1	Tax_Id=9606 Gene_Symbol=F2 Prothrombin (Fragment)	-
6.791482	-6.508139	-6.620306	-6.484527	-6.381975	-6.442644	-
5.800154	-6.929427	-6.522359	-6.155801	-6.007773	-6.820155	32
34	36	39	59	26	55	20	43	25	34	18
IPI00021304.1	"Tax_Id=9606 Gene_Symbol=KRT2 Keratin, type II
cytoskeletal 2 epidermal"	-8.347618	-7.873585	-8.294225	-7.699492
-7.856766	-6.21069	-7.358891	-8.16971	-8.710431	-7.108402
-6.626005	-6.802398	7	9	7	12	14	34	12	6
5	10	19	19
IPI00021727.1	Tax_Id=9606 Gene_Symbol=C4BPA C4b-binding protein alpha
chain -6.089061	-6.164835	-6.356139	-5.932678	-6.074685	-5.853055
-5.57681	-6.146467	-6.687188	-5.966358	-5.62191	-5.931834
62	46	45	65	77	45	66	42	35	29	48	42
IPI00947496.1	Tax_Id=9606 Gene_Symbol=- 124 kDa protein	-7.314527
-7.091809	-7.491658	-7.596264	-7.487834	-7.57636	-6.702278
-7.250733	-7.976857	-7.125133	-6.93975	-7.349758	34	34
27	23	35	15	40	26	18	17	24	19
IPI00017601.1	Tax_Id=9606 Gene_Symbol=CP Ceruloplasmin	-7.536912
-7.353414	-7.606121	-7.795506	-7.386385	-7.530481	-7.349545
-7.518511	-7.87691	-7.609882	-7.432867	-7.683368	26	25
23	18	37	15	20	19	19	10	14	13

IPI00019359.4	"Tax_Id=9606 Gene_Symbol=KRT9 Keratin, type I cytoskeletal 9"	-7.956239	-8.090196	-10.205431	-9.456547	-9.362507	-6.483471	-7.729652	-8.317328	-9.186553	-7.296841	-6.896683	-6.821762	10	7	1	2	3	25	8	5	3	8	14	18
IPI00785084.2	Tax_Id=9606 Gene_Symbol=LOC100294459;IGHV4-31;IGHG1;LOC100290146 IGH@ protein	-3.819986	-3.326865	-3.514327	-3.374078	-3.319544	-3.841494	-3.605788	-3.681014	-3.37793	-3.010729	-3.429101	-3.372252	467	612	601	654	943	262	369	385	746	434	335	423
IPI00816314.1	Tax_Id=9606 Gene_Symbol=LOC100294459;IGHV4-31;IGHG1;LOC100290146 Putative uncharacterized protein DKFZp686I15196	-3.830737	-3.333927	-3.519809	-3.379289	-3.319574	-3.851305	-3.627089	-3.685763	-3.378739	-3.012878	-3.431249	-3.386291	463	609	599	652	945	260	362	384	747	434	335	418
IPI00641737.1	Tax_Id=9606 Gene_Symbol=HP Haptoglobin	-4.671576	-5.482931	-4.478421	-4.857563	-4.183505	-4.743707	-4.741973	-4.869035	-5.413537	-5.763115	-4.266251	-4.654402	180	64	207	134	359	96	107	106	88	25	131	106
IPI00892870.1	Tax_Id=9606 Gene_Symbol=IGHM Protein	-5.866137	-6.065413	-5.812744	-5.379244	-4.702842	-5.157853	-5.863719	-5.202301	-5.492492	-5.597962	-4.949862	-5.404968	61	40	61	89	239	71	39	85	91	33	74	56
IPI00784842.1	Tax_Id=9606 Gene_Symbol=LOC100294459;IGHV4-31;IGHG1;LOC100290146 Putative uncharacterized protein DKFZp686G11190	-3.845555	-3.349777	-3.533942	-3.393828	-3.338703	-3.862771	-3.640708	-3.699697	-3.399208	-3.032007	-3.438509	-3.398269	465	611	602	655	945	262	364	386	746	434	339	421
IPI00876888.1	Tax_Id=9606 Gene_Symbol=LOC100294459;IGHV4-31;IGHG1;LOC100290146 cDNA FLJ78387	-3.841372	-3.345079	-3.529269	-3.387492	-3.335547	-3.864098	-3.639882	-3.693361	-3.387524	-3.025671	-3.441062	-3.391933	464	610	601	655	942	260	362	386	750	434	336	421
IPI00384938.1	Tax_Id=9606 Gene_Symbol=LOC100294459;IGHV4-31;IGHG1;LOC100290146 Putative uncharacterized protein DKFZp686N02209	-3.864495	-3.367685	-3.551899	-3.414583	-3.351218	-3.881224	-3.660847	-3.714326	-3.412498	-3.046636	-3.462027	-3.408159	463	609	600	651	947	261	362	386	747	434	336	423
IPI00479708.6	Tax_Id=9606 Gene_Symbol=IGHM Full-length cDNA clone CS0DD006YL02 of Neuroblastoma of Homo sapiens	-5.819378	-5.944967	-5.727519	-5.365408	-4.755002	-5.187393	-5.900276	-5.17065	-5.666671	-5.536458	-4.900986	-5.354365	51	36	53	72	181	55	30	70	61	28	62	47
IPI00739237.1	Tax_Id=9606 Gene_Symbol=LOC653879 similar to complement component 3	-5.499965	-5.363014	-5.805206	-5.749469	-5.48186	-5.496278	-4.874786	-5.154301	-5.773714	-6.07467	-5.511993	-5.947897	73	67	51	51	91	42	87	74	57	17	35	27
IPI00292530.1	Tax_Id=9606 Gene_Symbol=ITIH1 Inter-alpha-trypsin inhibitor heavy chain H1	-6.788673	-6.919595	-6.778765	-6.745501	-7.034453	-7.037821	-6.42789	-6.751414	-7.138801	-6.424075	-6.389376	-6.724834	47	33	45	44	45	21	43	35	34	28	34	29

IPI00009865.4	"Tax_Id=9606 Gene_Symbol=KRT10 Keratin, type I cytoskeletal 10"	-7.709272	-8.362023	-8.194876	-7.887824	-8.199249
	-6.171966	-6.006779	-8.070361	-7.447931	-7.009052	-6.212998
	-7.082538	12	5	7	9	9	32	42	6	16	10
	26	13
IPI00879709.3	Tax_Id=9606 Gene_Symbol=C6 Complement component 6 precursor	-7.840131	-8.371184	-8.674041	-8.166319	-8.477744	-
8.170957	-7.279175	-8.731848	-8.134736	-8.404508	-7.870819	-
7.824068	17	8	7	11	11	7	19	5	13	4	8
	10
IPI00645363.2	Tax_Id=9606 Gene_Symbol=LOC100294459;IGHV4-31;IGHG1;LOC100290146 Putative uncharacterized protein DKFZp686P15220
	-3.84353	-3.34672	-3.532602	-3.392083	-3.335547	-3.864098
	-3.639882	-3.698556	-3.392872	-3.025671	-3.444042	-3.399084
	463	609	599	652	942	260	362	384	746	434	335	418
IPI00386879.1	"Tax_Id=9606 Gene_Symbol=IGHA1 cDNA FLJ14473 fis, clone MAMMA1001080, highly similar to Homo sapiens SNC73 protein (SNC73) mRNA"
	-4.277421	-5.434647	-5.309981	-5.774549	-4.795386	-5.538511
	-5.099746	-5.079634	-6.315485	-5.560753	-5.333438	-5.491138
	314	79	106	63	229	51	88	101	42	36	53	54
IPI00305461.4	"Tax_Id=9606 Gene_Symbol=ITIH2 Inter-alpha (Globulin) inhibitor H2, isoform CRA_a"	-7.151217	-6.989123	-7.328347	-7.523925
	-7.242286	-7.287886	-6.315824	-6.734601	-7.371713	-6.57616
	-6.818999	-7.086363	34	32	27	21	38	17	50	37
	28	25	23	21
IPI00022488.1	Tax_Id=9606 Gene_Symbol=HPX Hemopexin	-6.04782	-
5.694073	-5.881098	-6.295365	-5.831404	-5.642165	-5.399239	-
5.602433	-6.375266	-5.676104	-5.710398	-5.886791	50	57	56
	35	76	43	61	56	37	30	34	34
IPI00022895.7	Tax_Id=9606 Gene_Symbol=A1BG Alpha-1B-glycoprotein	-
6.850782	-6.30961	-6.797389	-6.661609	-6.70477	-6.336864	-
6.360229	-6.518723	-7.221963	-6.055252	-6.472538	-6.709556	24
	33	24	26	34	23	25	24	17	22	17	16
IPI00026314.1	Tax_Id=9606 Gene_Symbol=GSN Isoform 1 of Gelsolin	-
7.847075	-7.044538	-7.488301	-7.979108	-7.469552	-7.444749	-
6.900908	-7.158342	-7.568034	-7.406367	-6.929835	-7.454535	14
	25	19	11	25	12	23	20	19	9	17	12
IPI00291867.3	Tax_Id=9606 Gene_Symbol=CFI Complement factor I	-
7.79457	-7.571851	-7.500015	-7.038813	-7.450321	-7.556546	-
6.484638	-7.221349	-7.174283	-6.825017	-6.696792	-8.036336	11
	11	14	21	19	8	26	14	21	12	16	5
IPI00296608.6	Tax_Id=9606 Gene_Symbol=C7 Complement component C7	-
8.615335	-7.773577	-8.109956	-8.14953	-7.505443	-8.058857	-
8.165604	-7.395973	-8.795826	-7.886944	-7.758719	-8.222794	7
	13	11	10	26	7	7	17	6	6	8	6
IPI00022395.1	Tax_Id=9606 Gene_Symbol=C9 Complement component C9	-
7.665521	-8.541415	-8.017593	-7.643402	-7.26168	-7.514509	-
7.21579	-7.420474	-8.230858	-11.100467	-7.635584	-7.994298	12
	4	8	11	22	8	12	11	7	0	6	5
IPI00553177.1	Tax_Id=9606 Gene_Symbol=SERPINA1 Isoform 1 of Alpha-1-antitrypsin	-4.689275	-4.18172	-4.916017	-5.228841	-4.239008	-
4.51579	-4.233879	-4.312765	-4.762136	-4.728723	-4.962288	-
4.364308	176	234	133	92	338	120	177	184	168	70	65
	141

IPI00647704.1 "Tax_Id=9606 Gene_Symbol=IGHA1 cDNA FLJ41552 fis, clone COLON2004478, highly similar to Protein Tro alpha1 H,myeloma" -4.264762 -5.409646 -5.282072 -5.743296 -4.795386 -5.558313 -5.088447 -5.021926 -6.364275 -5.560753 -5.333438 -5.419679 318 81 109 65 229 50 89 107 40 36 53 58
IPI00449920.1 "Tax_Id=9606 Gene_Symbol=IGHA1 cDNA FLJ90170 fis, clone MAMMA1000370, highly similar to Ig alpha-1 chain C region" -4.275394 -5.432621 -5.327003 -5.788523 -4.780345 -5.556287 -5.08642 -5.077608 -6.362248 -5.558727 -5.276351 -5.452744 314 79 104 62 232 50 89 101 40 36 56 56
IPI00423462.5 Tax_Id=9606 Gene_Symbol=IGHA1 Putative uncharacterized protein DKFZp686K18196 (Fragment) -4.326789 -5.484015 -5.368828 - 5.839918 -4.844755 -5.607682 -5.160543 -5.129003 -6.413643 - 5.610121 -5.382806 -5.540507 314 79 105 62 229 50 87 101 40 36 53 54
IPI00385264.1 Tax_Id=9606 Gene_Symbol=- Ig mu heavy chain disease protein -6.237637 -6.27443 -6.050713 -5.556721 -5.075299 - 5.598922 -6.124379 -5.453594 -6.058126 -6.020072 -5.332232 - 5.662775 35 27 40 62 137 38 25 55 43 18 42 36
IPI00784830.1 "Tax_Id=9606 Gene_Symbol=LOC100126583;IGHA2 cDNA FLJ41981 fis, clone SMINT2011888, highly similar to Protein Tro alpha1 H,myeloma" -5.137345 -6.355836 -6.174959 -6.18984 -5.66096 - 5.929704 -5.712664 -6.154123 -7.266294 -6.134268 -6.153964 - 6.693262 131 31 44 41 95 34 47 34 16 20 23 16
IPI00940245.1 Tax_Id=9606 Gene_Symbol=LOC100126583;IGHA2 Immunoglobulin heavy chain variant (Fragment) -4.979766 -6.198257 - 6.01738 -6.056954 -5.524659 -5.743138 -5.576591 -6.026397 - 7.108715 -5.97669 -5.996385 -6.600222 131 31 44 40 93 35 46 33 16 20 23 15
IPI00426051.3 Tax_Id=9606 Gene_Symbol=IGHG2 Putative uncharacterized protein DKFZp686C15213 -4.496102 -4.252506 -4.540131 -4.416953 - 4.391906 -4.941777 -4.464576 -4.828083 -4.453169 -4.176346 - 4.635908 -4.49749 237 242 215 230 322 87 156 122 254 135 100 137
IPI00477597.2 Tax_Id=9606 Gene_Symbol=HPR Isoform 1 of Haptoglobin-related protein -5.051508 -5.842844 -5.007967 -5.101442 -4.861495 -5.042466 -4.978255 -5.067756 -5.732529 -6.154882 -4.842523 -5.178546 102 37 101 87 151 59 70 72 53 14 61 52
IPI00785067.1 Tax_Id=9606 Gene_Symbol=LOC100126583;IGHA2 IGH@ protein -5.122867 -6.374148 -6.160481 -6.200055 -5.667759 -5.886239 -5.719692 -6.169498 -7.251816 -6.11979 -6.139486 -6.743323 131 30 44 40 93 35 46 33 16 20 23 15
IPI00032179.3 Tax_Id=9606 Gene_Symbol=SERPINC1 Antithrombin-III - 6.91964 -6.186096 -6.509572 -6.358525 -6.405257 -6.272191 - 5.563188 -5.606752 -6.771627 -5.990579 -6.245346 -6.421739 21 35 30 33 43 23 52 56 25 22 20 20
IPI00296165.6 "Tax_Id=9606 Gene_Symbol=C1R cDNA FLJ54471, highly similar to Complement C1r subcomponent" -7.4577 -6.960545 -7.304224 -6.997173 -6.993516 -7.76622 -6.733533 -6.811984 -6.994493 -6.523866 -6.971005 -7.022235 19 25 21 27 37 8 25 26 31 20 15 17

IPI00298971.1 Tax_Id=9606 Gene_Symbol=VTN Vitronectin -7.103517 -6.635676 -6.996057 -7.051545 -6.900347 -6.441679 -6.142961 -7.096881 -7.07579 -6.914123 -6.326366 -6.962291 18 23 19 17 27 20 30 13 19 9 19 12
IPI00296099.6 Tax_Id=9606 Gene_Symbol=THBS1 Thrombospondin-1 -9.790425 -9.567706 -9.226206 -9.170469 -8.788747 -10.33256 -9.746159 -8.254393 -9.30594 -11.839077 -11.998534 -9.243734 3 3 5 5 10 1 2 10 5 0 0 3
IPI00827754.3 Tax_Id=9606 Gene_Symbol=IGHG3 Ig gamma-3 chain C region -3.824278 -3.53239 -3.752487 -3.544835 -3.588917 -4.437872 -4.018526 -4.096588 -3.783927 -3.692198 -3.710429 -3.708574 377 404 384 447 584 117 198 206 403 178 205 245
IPI00399007.7 Tax_Id=9606 Gene_Symbol=IGHG2 Putative uncharacterized protein DKFZp686I04196 (Fragment) -3.891217 -3.832527 -3.998836 -3.873304 -4.021788 -4.257462 -3.995988 -4.36052 -4.16012 -3.738381 -4.251478 -3.935395 390 331 332 356 419 155 224 175 306 188 132 216
IPI00847635.1 Tax_Id=9606 Gene_Symbol=SERPINA3 Isoform 1 of Alpha-1-antichymotrypsin -6.021503 -5.842269 -6.234738 -6.048948 -5.854437 -5.731654 -5.361477 -5.396457 -5.820453 -5.275537 -5.410896 -5.496318 47 45 36 41 68 36 58 63 59 41 42 46
IPI00555812.4 Tax_Id=9606 Gene_Symbol=GC Isoform 1 of Vitamin D-binding protein -6.618189 -6.395471 -6.321174 -6.475158 -7.096738 -6.061712 -6.400261 -6.434551 -6.753729 -6.011901 -6.777495 -6.666205 29 29 37 30 22 29 23 25 26 22 12 16
IPI00644018.1 Tax_Id=9606 Gene_Symbol=A1BG 41 kDa protein -7.037806 -6.432095 -6.802091 -6.803513 -6.770097 -6.356166 -6.523537 -6.641209 -7.28729 -6.030102 -6.383715 -6.560108 15 22 18 17 24 17 16 16 12 17 14 14
IPI00017696.1 Tax_Id=9606 Gene_Symbol=C1S Complement C1s subcomponent -8.748629 -8.189438 -8.358763 -8.862643 -8.162466 -7.604365 -7.343387 -7.723423 -8.438497 -11.308107 -7.843223 -7.865466 5 7 7 4 11 9 13 10 7 0 6 7
IPI00303963.1 Tax_Id=9606 Gene_Symbol=C2 Complement C2 (Fragment) -8.837576 -8.432536 -9.007327 -9.644737 -8.85755 -9.890537 -7.800059 -10.114956 -8.393913 -7.485031 -9.030783 -9.207176 5 6 4 2 6 1 9 1 8 8 2 2
IPI00299145.9 "Tax_Id=9606 Gene_Symbol=KRT6C Keratin, type II cytoskeletal 6C" -8.773038 -11.769195 -11.93852 -8.663908 -12.194209 -7.656945 -8.323307 -9.134127 -10.185673 -7.667353 -7.356807 -8.514029 4 0 0 4 0 7 4 2 1 5 8 3
IPI00300725.7 "Tax_Id=9606 Gene_Symbol=KRT6A Keratin, type II cytoskeletal 6A" -8.549894 -11.769195 -11.93852 -9.357055 -12.194209 -7.656945 -8.323307 -9.134127 -10.185673 -7.667353 -7.490338 -8.919494 5 0 0 2 0 7 4 2 1 5 7 2
IPI00930442.1 Tax_Id=9606 Gene_Symbol=IGHG4 Putative uncharacterized protein DKFZp686M24218 -4.151965 -4.097096 -4.22257 -4.093669 -4.392898 -4.662534 -4.35258 -4.715995 -4.417615 -4.083274 -4.487488 -4.1397 343 290 303 326 330 118 179 140 270 152 119 201

IPI00922744.1 Tax_Id=9606 Gene_Symbol=C4B Complement protein C4B frameshift mutant (Fragment) -6.138227 -6.905907 -6.595659 -6.694073
-6.1582 -5.959044 -5.632927 -5.960319 -6.886702 -6.508448
-6.137277 -6.661977 35 13 21 18 42 24 37 30
17 10 17 12
IPI00032328.2 Tax_Id=9606 Gene_Symbol=KNG1 Isoform HMW of Kininogen-1
-7.296244 -6.773421 -7.06053 -6.815551 -7.6039 -6.902286
-6.441049 -7.069546 -7.833411 -6.770378 -6.57316 -7.106228
20 27 24 29 18 17 30 18 12 14 20 14
IPI00879573.1 Tax_Id=9606 Gene_Symbol=SERPIND1 Heparin cofactor 2 -
7.839659 -7.868255 -9.290344 -7.981844 -7.531129 -7.534497 -
6.696782 -7.219919 -8.271465 -8.05573 -7.522041 -7.092298 9
7 2 7 15 7 18 12 6 3 6 11
IPI00294395.1 Tax_Id=9606 Gene_Symbol=C8B Complement component C8 beta chain -8.414335 -8.373938 -8.543263 -8.71067 -8.616629 -7.703707
-7.191414 -9.180888 -8.84614 -11.156134 -7.873572 -7.867643
6 5 5 4 6 7 13 2 4 0 5 6
IPI00294004.1 Tax_Id=9606 Gene_Symbol=PROS1 Vitamin K-dependent protein S -8.26103 -9.01914 -8.207636 -9.132729 -8.751006 -
7.992234 -7.811299 -8.216653 -8.980517 -9.45793 -8.231093 -
8.407486 8 3 8 3 6 6 8 6 4 1 4
4
IPI00478809.3 Tax_Id=9606 Gene_Symbol=F5 Coagulation factor V -
10.145046 -10.21001 -10.7848 -11.422211 -10.124198 -12.807445 -
9.289851 -10.10067 -10.459069 -9.550187 -10.808257 -9.19289 4
3 2 1 5 0 6 3 3 3 1 6
IPI00021841.1 Tax_Id=9606 Gene_Symbol=APOA1 Apolipoprotein A-I -
6.772469 -6.097766 -6.222639 -6.81749 -5.64353 -5.636173 -
5.783742 -5.495949 -6.729817 -6.449544 -6.385857 -6.092247 14
22 23 12 53 25 24 36 15 8 10 16
IPI00298828.3 Tax_Id=9606 Gene_Symbol=APOH Beta-2-glycoprotein 1 -
7.269927 -6.672515 -6.975372 -6.919635 -7.097529 -6.71345 -
6.820197 -6.850857 -6.986113 -7.398987 -6.865297 -6.923907 11
16 14 14 16 11 11 12 15 4 8 9
IPI00654888.4 Tax_Id=9606 Gene_Symbol=KLKB1 Plasma kallikrein -
8.490857 -7.494948 -8.031998 -7.608537 -8.693152 -7.780229 -
7.347979 -8.004648 -8.699519 -7.790637 -7.362308 -7.790015 6
13 9 13 6 7 12 7 5 5 9 7
IPI00293665.9 "Tax_Id=9606 Gene_Symbol=KRT6B Keratin, type II cytoskeletal 6B" -8.367573 -11.769195 -11.93852 -8.663908 -9.66848
-7.523413 -7.917842 -9.134127 -10.185673 -7.890496 -7.490338
-8.003203 6 0 0 4 2 8 6 2 1 4
7 5
IPI00889156.1 Tax_Id=9606 Gene_Symbol=IGKV3-20 IGK@ protein -3.087601
-3.466969 -3.23578 -3.505657 -3.590507 -3.379774 -3.994192
-3.307077 -3.209657 -4.115109 -3.777534 -3.576633 493 270
403 291 365 211 127 284 448 73 120 175
IPI00894384.1 Tax_Id=9606 Gene_Symbol=LOC100126583;IGHA2 Putative uncharacterized protein DKFZp686O16217 (Fragment) -5.34353 -6.721117
-6.484977 -6.747694 -5.960507 -6.482668 -6.289311 -7.063762
-7.663323 -6.444286 -6.367355 -7.090292 109 22 33 24
72 20 27 14 11 15 19 11
IPI00291866.5 Tax_Id=9606 Gene_Symbol=SERPING1 Plasma protease C1 inhibitor -7.148514 -7.17711 -7.095121 -6.885234 -7.468592 -

6.263533	-6.187958	-6.30563	-7.58032	-6.383756	-7.369892	-
8.105901	18	14	18	21	16	25	30	30	12	16	7
	4
IPI00947137.1	Tax_Id=9606 Gene_Symbol=- Protein	-6.83654	-6.431499
	-6.783146	-6.602247	-6.856513	-6.908671	-6.15042	-6.647582
	-7.62502	-6.264153	-5.825773	-6.600003	15	18	15	17
	18	8	19	13	7	11	20	11
IPI00006114.4	Tax_Id=9606 Gene_Symbol=SERPINF1 Pigment epithelium-
derived factor	-7.026546	-6.997984	-7.408471	-7.553405	-7.422997
	-8.610135	-6.151932	-7.042794	-7.806659	-7.185458	-6.571726
	-7.233627	17	14	11	9	14	2	26	12	8	6
	13	8
IPI00879231.1	Tax_Id=9606 Gene_Symbol=SERPINF2 Alpha-2-antiplasmin
	-7.941281	-7.313097	-7.664744	-7.513697	-7.658068	-8.07795
	-6.737778	-7.386078	-8.660768	-7.751886	-6.995053	-7.682271
	8	12	10	11	13	4	17	10	4	4	10	6
IPI00654755.3	Tax_Id=9606 Gene_Symbol=HBB Hemoglobin subunit beta	-
6.512125	-6.800233	-5.670275	-7.606968	-7.225246	-7.159621	-
5.725923	-6.690893	-8.147904	-6.545875	-6.482189	-6.658582	10
	6	22	3	6	3	14	6	2	4	5	5
IPI00021842.1	Tax_Id=9606 Gene_Symbol=APOE Apolipoprotein E	-9.58318
	-8.261849	-6.890729	-7.68229	-7.387579	-6.461753	-7.187539
	-8.152509	-8.510908	-7.314344	-10.692677	-10.86907	1	3
	14	6	11	13	7	3	3	4	0	0
IPI00011252.1	Tax_Id=9606 Gene_Symbol=C8A Complement component C8
alpha chain	-8.248269	-8.585166	-9.042173	-8.698755	-8.317032	-
8.028264	-7.346553	-8.475826	-8.42876	-11.144219	-7.679335	-
7.855728	7	4	3	4	8	5	11	4	6	0	6
	6
IPI00020996.5	Tax_Id=9606 Gene_Symbol=IGFALS Insulin-like growth
factor-binding protein complex acid labile subunit	-12.062088	-8.620494
	-9.482966	-9.427229	-8.640042	-7.727119	-7.582551	-8.511154
	-8.309937	-8.653818	-8.120128	-9.682815	0	4	2	2
	6	7	9	4	7	2	4	1
IPI00023014.2	Tax_Id=9606 Gene_Symbol=VWF von Willebrand factor	-
11.766285	-13.376148	-11.712892	-13.489736	-9.889138	-13.042389	-
10.217942	-11.434226	-11.099478	-12.716325	-9.251441	-13.052175	1
	0	1	0	8	0	3	1	2	0	6	0
IPI00784865.1	Tax_Id=9606 Gene_Symbol=IGK@ IGK@ protein	-3.137507
	-3.508563	-3.258366	-3.533533	-3.626773	-3.408622	-4.084789
	-3.365064	-3.363808	-4.278434	-3.820094	-3.60562	469	259
	394	283	352	205	116	268	384	62	115	170
IPI00154742.6	Tax_Id=9606 Gene_Symbol=IGLV2-14;IGLC2 IGL@ protein	-
4.275653	-4.34735	-4.621036	-4.826664	-4.277887	-4.304281	-
4.059184	-4.29358	-4.651979	-4.635858	-5.060007	-4.158197	149
	111	100	77	182	83	118	105	105	43	33	97
IPI00893853.1	Tax_Id=9606 Gene_Symbol=LOC100126583;IGHA2 Protein	-
4.744198	-6.130918	-5.864925	-6.077452	-5.442281	-5.99783	-
5.593751	-6.279407	-6.986113	-5.728924	-5.777156	-6.25893	110
	22	34	26	67	18	30	17	12	17	19	14
IPI00423461.3	Tax_Id=9606 Gene_Symbol=LOC100126583;IGHA2 Putative
uncharacterized protein DKFZp686C02220 (Fragment)	-5.356755	-6.725125
	-6.488985	-6.751702	-5.992686	-6.486676	-6.293319	-7.06777

-7.500277 -6.517287 -6.371363 -7.007288 108 22 33 24
70 20 27 14 13 14 19 12
IPI00032220.3 Tax_Id=9606 Gene_Symbol=AGT Angiotensinogen -7.52352
-6.489871 -6.959301 -6.680421 -6.991846 -7.372508 -7.160801
-6.275171 -7.201554 -7.046444 -6.289611 -6.571364 12 27
20 25 25 8 11 30 17 8 20 18
IPI00291262.3 Tax_Id=9606 Gene_Symbol=CLU Isoform 1 of Clusterin -
7.158712 -7.223676 -7.575323 -7.049582 -7.243224 -7.072238 -
6.303517 -6.55472 -7.185053 -6.340709 -6.643267 -6.126513 16
12 10 16 18 10 24 21 16 15 13 26
IPI00022426.1 Tax_Id=9606 Gene_Symbol=AMBP Protein AMBP -7.290014
-6.631977 -6.744144 -6.806191 -7.056991 -6.646525 -6.673229
-6.870944 -7.075192 -7.419073 -6.56693 -6.502161 11 17
18 16 17 12 13 12 14 4 11 14
IPI00218732.3 Tax_Id=9606 Gene_Symbol=PON1 Serum
paraoxonase/arylesterase 1 -7.131446 -7.171092 -7.340417 -7.284681
-7.259633 -6.837333 -6.474077 -7.167113 -7.324841 -10.646436
-7.181552 -7.070263 13 10 10 10 14 10 16 9
11 0 6 8
IPI00011261.2 Tax_Id=9606 Gene_Symbol=C8G Complement component C8
gamma chain -7.053104 -7.300389 -7.133242 -6.720831 -7.38893 -
6.273483 -6.197909 -6.854577 -7.212976 -6.86371 -7.023167 -
7.487242 8 5 7 10 7 10 12 7 7 4 4
3
IPI00163207.1 Tax_Id=9606 Gene_Symbol=PGLYRP2 Isoform 1 of N-
acetylmuramoyl-L-alanine amidase -7.877801 -8.011757 -8.181082 -
8.684961 -7.984785 -7.677998 -7.42807 -7.768886 -8.597288 -
8.604697 -7.665542 -7.841935 10 7 7 4 11 7 10
8 5 2 6 6
IPI00953689.1 Tax_Id=9606 Gene_Symbol=AHSG Alpha-2-HS-glycoprotein
-8.631028 -8.813774 -8.983099 -8.234216 -8.140175 -7.563725
-7.082685 -8.298969 -9.75598 -8.153951 -8.313409 -8.084336
3 2 2 4 6 5 9 3 1 2 2 3
IPI00304273.2 Tax_Id=9606 Gene_Symbol=APOA4 Apolipoprotein A-IV -
9.112545 -7.973536 -7.96054 -8.310268 -8.621693 -11.081796 -
7.410052 -7.527724 -11.664614 -10.755732 -10.91519 -11.091583 2
5 6 4 4 0 7 7 0 0 0 0
IPI00550731.2 Tax_Id=9606 Gene_Symbol=- Putative uncharacterized
protein -3.139534 -3.475904 -3.258387 -3.518288 -3.616932 -
3.406726 -4.063519 -3.366564 -3.360935 -4.141534 -3.749344 -
3.606556 474 271 399 291 360 208 120 271 390 72 125
172
IPI00784773.13 Tax_Id=9606 Gene_Symbol=- Putative uncharacterized
protein -3.152545 -3.520214 -3.276292 -3.562209 -3.623937 -
3.423365 -4.137899 -3.383897 -3.36903 -4.311224 -3.846527 -
3.635473 462 256 387 275 353 202 110 263 382 60 112
165
IPI00853045.1 Tax_Id=9606 Gene_Symbol=IGKC Anti-RhD monoclonal T125
kappa light chain -3.144034 -3.511703 -3.267781 -3.553698 -3.626822
-3.414854 -4.120338 -3.375387 -3.36314 -4.269923 -3.838016
-3.626962 462 256 387 275 349 202 111 263 381 62
112 165
IPI00909649.4 Tax_Id=9606 Gene_Symbol=IGKC Ig kappa chain C region
-3.543785 -3.911454 -3.667532 -3.953449 -4.023712 -3.814605

-4.529139 -3.775137 -3.762891 -4.702464 -4.237767 -4.026713
462 256 387 275 350 202 110 263 381 60 112 165
IPI00956314.2 Tax_Id=9606 Gene_Symbol=IGLC3 hypothetical protein
XP_002348153 -4.4444 -4.50468 -4.869497 -5.042602 -4.409565
-4.526262 -4.205564 -4.493458 -4.801595 -4.780016 -5.309847
-4.320936 142 107 88 70 180 75 115 97 102 42
29 93
IPI00887169.2 Tax_Id=9606 Gene_Symbol=IGLV1-44 Putative
uncharacterized protein -4.325265 -4.401952 -4.827997 -4.822271
-4.354606 -4.349606 -4.110992 -4.391703 -4.739739 -4.667899
-5.19773 -4.298767 143 106 82 78 170 80 113 96
97 42 29 85
IPI00807428.1 Tax_Id=9606 Gene_Symbol=- Putative uncharacterized
protein -4.342221 -4.446024 -4.926405 -4.940627 -4.298773 -
4.45072 -4.106745 -4.430016 -4.799345 -4.663652 -5.382726 -
4.294521 140 101 74 69 179 72 113 92 91 42 24
85
IPI00292946.1 Tax_Id=9606 Gene_Symbol=SERPINA7 Thyroxine-binding
globulin -7.36765 -6.739466 -7.401268 -7.797516 -7.489902 -
7.350169 -7.100241 -7.122603 -7.932987 -7.178256 -7.337713 -
7.919571 12 18 11 7 13 7 10 11 7 6 6
4
IPI00025204.1 Tax_Id=9606 Gene_Symbol=CD5L CD5 antigen-like -7.881843
-7.841446 -7.82845 -7.772713 -7.678673 -7.037684 -7.144431
-7.732106 -7.620502 -8.097914 -6.753294 -7.181001 6 5
6 6 9 8 8 5 8 2 9 7
IPI00022391.1 Tax_Id=9606 Gene_Symbol=APCS Serum amyloid P-component
-6.928865 -7.622437 -7.232146 -8.023708 -7.035849 -6.372387
-6.479134 -7.513097 -7.648352 -7.655761 -7.122071 -7.991611
10 4 7 3 11 10 10 4 5 2 4 2
IPI00909594.1 "Tax_Id=9606 Gene_Symbol=C7 cDNA FLJ58413, highly
similar to Complement component C7" -8.624192 -7.708327 -8.011183
-8.515062 -7.910196 -8.067715 -7.951318 -7.732518 -8.650533
-7.741651 -7.677965 -8.770648 4 8 7 4 10 4
5 7 4 4 5 2
IPI00328609.3 Tax_Id=9606 Gene_Symbol=SERPINA4 Kallistatin -8.494768
-7.712433 -9.134522 -8.385638 -8.291597 -9.324585 -6.946425
-7.757244 -8.115643 -8.305373 -7.078536 -7.724933 4 7
2 4 6 1 12 6 6 2 8 5
IPI00383338.1 Tax_Id=9606 Gene_Symbol=ITIH1 PRO2769 -7.628918 -
8.417801 -8.874808 -7.720459 -8.842814 -8.084042 -7.179188 -
9.001608 -8.66686 -10.976853 -7.917435 -7.688363 11 4 3
9 4 4 11 2 4 0 4 6
IPI00479116.1 Tax_Id=9606 Gene_Symbol=CPN2 Carboxypeptidase N subunit
2 -9.431917 -8.292907 -10.071671 -8.406496 -8.247917 -8.469974
-8.065895 -9.793005 -9.052792 -11.075104 -7.792542 -7.968935
2 5 1 5 8 3 5 1 3 0 5 5
IPI00019943.1 Tax_Id=9606 Gene_Symbol=AFM Afamin -9.120927 -8.205062
-7.863561 -11.942991 -9.323222 -8.56445 -8.383515 -9.194334
-10.24588 -11.16958 -8.397843 -8.286554 3 6 10 0
3 3 4 2 1 0 3 4
IPI00022371.1 Tax_Id=9606 Gene_Symbol=HRG Histidine-rich glycoprotein
-8.989064 -8.478663 -8.935671 -8.879934 -8.680533 -7.739439

-8.539333 -9.062471 -9.42087 -11.037716 -8.671445 -8.154691
3 4 3 3 5 6 3 2 2 0 2 4
IPI00009867.3 "Tax_Id=9606 Gene_Symbol=KRT5 Keratin, type II
cytoskeletal 5" -8.412641 -11.814263 -11.983589 -10.095271 -10.406695
-7.856164 -9.061523 -9.179195 -10.230741 -8.223247 -7.689557
-8.964562 6 0 0 1 1 6 2 2 1 3
6 2
IPI00746623.2 Tax_Id=9606 Gene_Symbol=HABP2 Hyaluronan-binding protein
2 -9.459068 -9.929496 -9.405674 -8.944473 -12.187091 -11.428319
-8.093046 -8.433862 -9.079943 -8.576526 -8.330518 -8.506911
2 1 2 3 0 0 5 4 3 2 3 3
IPI00007240.2 Tax_Id=9606 Gene_Symbol=F13B Coagulation factor XIII B
chain -9.624885 -11.927895 -9.571491 -9.515755 -8.728567 -11.594136
-8.769689 -8.599679 -12.176954 -8.742343 -8.496335 -9.771341
2 0 2 2 6 0 3 4 0 2 3 1
IPI00020986.2 Tax_Id=9606 Gene_Symbol=LUM Lumican -8.954177 -
9.424605 -11.426512 -8.439582 -8.463324 -9.090847 -7.405834 -
8.216653 -9.673665 -8.764782 -10.756821 -9.100633 2 1 0
3 4 1 6 3 1 1 0 1
IPI00242956.5 Tax_Id=9606 Gene_Symbol=FCGBP IgGFc-binding protein -
14.251939 -12.196639 -14.198546 -14.142809 -10.542211 -10.764268 -
11.969627 -13.919881 -11.752551 -11.536816 -13.528855 -11.872667 0
1 0 0 8 3 1 0 2 1 0 1
