# Supplementary material for: Serum Proteome Changes in Healthy Subjects with Different Genotypes of NOS1AP in the Chinese Population
Source: J Diabetes Res. 2013 Apr 7;2013:357630. doi: 10.1155/2013/357630 (PMC3647583; doi:10.1155/2013/357630)
Supplement: Supplementary file 2 [file 357630.f2.pdf]

Supplementary table2 Clinical characteristics of the CC carriers and AA carriers for validation

	CC (N=24)		AA (N=24)		P value
	mean	str	mean	str	
age (year)	56.92	1.55	57.46	1.17	0.7819
sex (male/female)	4/20		0/24		0.0428
BMI (kg/m2)	23.15	0.55	23.70	0.56	0.4889
FPG (mmol/L)	4.82	0.10	4.99	0.09	0.2331
2h glucose (mmol/L)	5.78	0.21	5.59	0.22	0.5365
HbA1c(%)	5.60	0.07	5.50	0.07	0.6238
TC (mmol/l)	4.93	0.21	5.19	0.19	0.3782
TG (mmol/L)	1.65	0.21	1.60	0.15	0.8425
HDL(mmol/L)	1.42	0.08	1.43	0.07	0.9649
LDL(mmol/L)	3.05	0.17	3.40	0.15	0.1408
